# *In Vitro* Study of Deuterium Effect on Biological Properties of Human Cultured Adipose-Derived Stem Cells

**DOI:** 10.1155/2018/5454367

**Published:** 2018-11-04

**Authors:** Alona Zlatska, Inna Gordiienko, Roman Vasyliev, Dmitriy Zubov, Olga Gubar, Anzhela Rodnichenko, Anton Syroeshkin, Igor Zlatskiy

**Affiliations:** ^1^State Institute of Genetic and Regenerative Medicine, National Academy of Medical Sciences of Ukraine, Kyiv, Ukraine; ^2^Biotechnology Laboratory Ilaya Regeneration, Medical Company Ilaya®, Kyiv, Ukraine; ^3^RE Kavetsky Institute of Experimental Pathology, Oncology and Radiobiology, National Academy of Sciences of Ukraine, Kyiv, Ukraine; ^4^Peoples Friendship University of Russia (RUDN University), 6 Miklukho-Maklaya St., Moscow 117198, Russia; ^5^Dumanskii Institute of Colloid Chemistry and Water Chemistry, National Academy of Sciences of Ukraine, Kyiv, Ukraine

## Abstract

In current* in vitro* study we have shown the impact of deuterium content in growth medium on proliferation rate of human cultured adipose-derived stem cells (ADSC). ADSCs have also demonstrated morphological changes when cultured in deuterated growth medium: the cell cultures did not reach confluence but acquired polygonal morphology with pronounced stress fibers. At high deuterium concentrations the ADSCs population doubling time increased which indicated the cell cycle retardation and decrease of cell proliferation rate. The deuterated and deuterium-depleted growth media demonstrated acute and chronic cytotoxicity, respectively. The minimal migration ability was observed in deuterated medium whereas the highest migration activity was observed in the medium with the deuterium content close to natural. The cells in deuterated growth medium demonstrated decrease in metabolic activity after three days in culture. In contrast, in deuterium-depleted medium there was an increase in ADSC metabolic activity.

## 1. Introduction

Natural water is a multicomponent mixture of molecules of different isotope composition. Depending on the protium/deuterium isotope ratio, the water changes its physicochemical properties [1]. However, the biological effect of deuterium has not been thoroughly studied. Deuterium-depleted water (ddw) has a number of unexpected biological properties, including antitumor [2], antidotal [3], and metabolic effects [4]. When protium (H) is completely replaced by deuterium (D), the 2-3-fold kinetic isotope effect is well known [5]. At the molecular level, it was found that the decrease in the deuterium content in water below the natural concentrations (<90 ppm) activates and reliably accelerates the respiratory chain reaction in the mitochondria, whereas in the excess of deuterium up to 99% this reaction is almost completely inhibited [6]. It was shown that different deuterium concentrations can variously affect the proliferation activity of the prokaryotic and eukaryotic cells* in vitro *[7–10]. Previously, a positive survival effect of deuterium-depleted water was demonstrated in assays with transplantable tumor cultures on laboratory animals and other antitumor properties have been demonstrated within experimental systems* in vivo *and* in vitro *[11–13].

Thus, the deuterium isotope content effect can activate or inhibit intracellular processes. However, until the primary information on deuterium toxicology is accumulated with proper* in vivo/in vitro *correlation (IVIVC) investigation, the human studies of its effect are impermissible.

Therefore, the aim of this study was to determine the effect of deuterium on biological properties of human adipose-derived stem cell (ADSCs) cultures within* in vitro *cell model.

## 2. Materials and Methods

### 2.1. Physicochemical Analysis of Water with Different Deuterium Content

The following basic water samples with different deuterium content were used in this study: deuterium-depleted water (ddw) with D/H= 4±2 ppm D (Sigma-Aldrich, USA); heavy water with D/H = 99 absolute at. % D (Sigma-Aldrich, USA). Growth media with various deuterium content were prepared by diluting deuterium-depleted and deuterated water. The following growth media were used in this study: No. 1: the medium with the highest deuterium content-D/H ratio 500.000 ppm; No. 2: medium with D/H ratio 100.000 ppm; No. 3: medium with D/H ratio 10.000 ppm; No. 4: D/H ratio 75 ppm; and No. 5: D/H ratio 15 ppm. The milliQ water served a standard with D/H = 150 ppm. MilliQ, deuterium-depleted and deuterated water had no differences in physical characteristic [14] or in trace element composition, except the deuterium content. This excluded the multifactor influence in the system for all comparison groups.

The deuterium content was controlled by multipass laser absorption spectroscopy on the Isotopic Water Analyzer-912-0032 (Los Gatos Research Inc., USA).

Chemical analysis of water with different deuterium content was performed by inductively coupled plasma-mass spectrometry on the ICP-QMS Agilent 7500CE spectrometer (Agilent Technologies, USA) [15]. Calibration solutions with a high range of elements' concentration (from 0.1 *μ*g/dm^3^to 100 *μ*g/dm^3^) were used for the device calibration. The solutions were prepared based on the international standard 2.74473.0100 “ICP Multi Element Standard Solution XXI CertiPUR*®*” which contains the following elements: Ag, Al, As, Ba, Be, Bi, Ca, Cd, Co, Cr, Cs, Cu, Fe, Ga, In, K, Li, Mg, Mn, Na, Ni, Pb, Rb, Se, Sr, Tl, U, V, Zn, and Hg. The concentration of all above-listed 24 elements in the milliQ, deuterium-depleted or heavy water did not exceed the upper detection limit (detection limit range – 0.1–10 ppm).

### 2.2. ADSCs Cell Culture

The experiments with use of human cell culture* in vitro *were carried out in accordance with the human experiment issues of the Code of Ethics of the World Medical Association (Declaration of Helsinki). In all cases the voluntary informed consent was signed by donors of ADSCs [16].

ADSCs were isolated from the lipoaspirate by enzymatic digestion in 0.1% collagenase IA and 0.1% pronase with 2% fetal bovine serum (FBS) (all from Sigma-Aldrich, USA) for 1 h at 37°С. The obtained cell suspension was transferred to 25 cm^2^ cell culture flask (SPL, Korea) and cultured in the following control growth medium: modified МЕМ-*α* (Sigma-Aldrich, USA) prepared from the powder diluting with milliQ water of natural isotope content supplemented with 10% FBS (Sigma-Aldrich, USA), 2mM L-glutamine, 100 U/ml penicillin, 100 *μ*g/ml streptomycin, and 1 ng/ml bFGFcon-2 (all from Sigma-Aldrich, USA).* Experimental growth media* had a composition similar to the control one but were prepared on the basis of deuterated and deuterium-depleted waters. The cells were cultured in multigas incubator CB210 (Binder, Germany) at +37°C in the atmosphere of saturated humidity, 5%  *СО*_2_ and 5%  *О*_2_.

To determine the effect of deuterium on the ADSCs biological properties, morphological changes, population doubling time, viability, migratory capacity, and proliferation rate of six cell lines at P2 were assessed, after 5 days of culturing.

### 2.3. Immunophenotype Analysis

The cell phenotype was assessed by fluorescence-activated cell sorting (FACS) on BD FACSAria flow cytometer (BD Pharmingen, BD Horizon USA). Staining with monoclonal antibodies (PerCP-Cy5.5 mouse antihuman CD105, APC mouse antihuman CD73, FITC mouse antihuman CD90, PE-Cy5 mouse antihuman HLA-DR, APC mouse antihuman CD34, and FITC mouse antihuman CD45) in accordance with manufacturer's instructions (BD Pharmingen, BD Horizon USA). The analysis was performed using BD FACS Diva 6.1 software (BD Pharmingen, BD Horizon USA).

### 2.4. ADSCs Morphology

ADSCs cell cultures were photographed in phase-contrast 24 hours and 72 hours after the control medium change for experimental medium. The presence of stress fibers, the number of mitoses, the size of the cells, and the granulation of the cytoplasm were registered in different comparison groups. The cultures were visualized and photos were taken with Carl Zeiss Axio ObserverA1 microscope, Axio Cam ERc 5s camera, and ZEN 2012 software (Carl Zeiss, Germany).

### 2.5. ADSCs Population Doubling Time

The population doubling time (PDT) was assessed at P2 using standard formula [17]:

PDT = T / 3.31 lg (X_k_ / X_0_)

where X_k_ is number of harvested cells; X_0_ is number of seeded cells; T is cell cultivation time.

The number of cells was counted with Goriaiev hemocytometer.

### 2.6. Cytotoxicity

ADSCs were seeded with a density of 1000 cells per 1 cm^2^. In 24 h the control growth medium was changed for the experimental one. For the cytotoxicity assessment the cells were stained with РI (propidium iodide) (Sigma-Aldrich, USA) and FDA (fluorescein diacetate) (Sigma-Aldrich, USA) after 24 h and 72 h in culture. PI does not penetrate into living cells; therefore it stains only those in which the integrity of the cell membrane was broken. FDA is not a fluorescent molecule, but when penetrated into living cells is converted into a fluorescent form, fluorescein. The number of dead and living ADSCs in different groups was counted using fluorescence microscopy (FITC and Texas Red filters; Carl Zeiss, Germany).

Cytotoxicity was calculated as the ratio of living cells to the total number of cells and was expressed as a percentage according to the formula:

Cytotoxicity= (total number of cells / number of living cells)×100%.

### 2.7. *In Vitro* Scratch Assay

ADSCs were seeded with a density of 1000 cells per 1 cm^2^. The medium was changed every 48 h. When the cell culture reached 90% confluence, the medium was changed for experimental one. The monolayer was scratched (~0.5 mm), the damaged area was marked, and photos were taken in 3, 24 and 48hrs. Migratory ability was calculated as the percentage of the scratch migration area to the area of damage. The area calculation was performed with ImageJ software (Wayne Rasband (NIH)).

### 2.8. ADSCs Metabolic Activity

ADSCs were seeded with a density of 1000 cells per 1 cm^2^. After the cells adhesion to plastic, the medium was changed for experimental one. In 24h and 72h 10% of Alamar Blue (redox indicator; Thermo Fisher, USA) was added to the culture medium and incubated for 3h [18]. Reduced Alamar Blue was detected at 540 nm versus 630 nm at Labsystems Multiskan PLUS spectrofluorimeter (USA). Cell metabolic activity was calculated according to the following formula:

% of reduction = ((*ε*ox) *λ*2·A*λ*1 - (*ε*ox) *λ*1·A*λ*2) experiment /((*ε*ox) *λ*2·A' *λ*1 - (*ε*ox) *λ*1· A' *λ*2 )control,

where *λ*1= 540 nm; *λ*2=630 nm; (*ε*ox) *λ*2 = 34,798; (*ε*ox) *λ*1 = 47,619; A*λ*1 is experimental sample absorption at *λ*1= 540 nm; A*λ*2 is experimental sample absorption at *λ*2= 630 nm; A' *λ*1 is control sample absorption at *λ*1= 540 nm; A' *λ*2 is control sample absorption at *λ*1= 630 nm.

### 2.9. Statistics

All statistical data processing was performed using Student's t-test in Microsoft Excel and Origin Pro. Differences between the comparison groups were considered reliable at p <0.05.

## 3. Results

### 3.1. Immunophenotype of Human ADSCs

**Figure 1:**
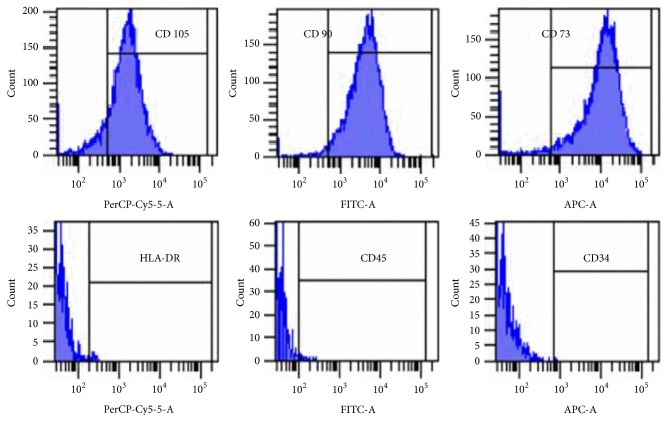
Representative FACS histograms.

 The results of the study of the immunophenotype of cell culture of the 2nd passage are presented in Table 1. Histograms in Figure 1 Illustrate flow cytometry results. ADSCs were analyzed on expression of the positive (CD73, CD90, and CD105) and negative (CD34, CD45, and HLA-DR) markers.

The flow cytometry analysis of the cell cultures showed the characteristic phenotype: CD73+CD90+CD105+CD34-CD45-HLA-DR- phenotype, which corresponds to the minimal criteria for the determination of ADSCs.

### 3.2. ADSCs Morphology Change after Culturing in Growth Media with Different Deuterium Content

**Figure 2:**
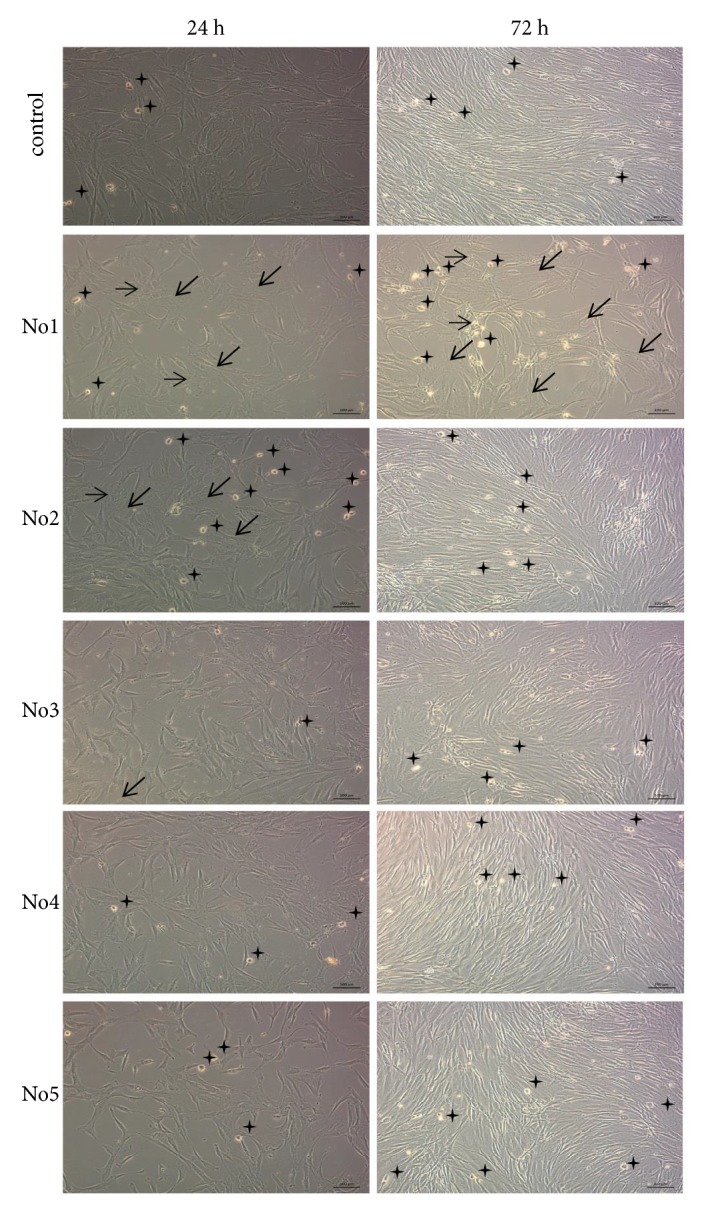
The morphology of ADSCs cultured in control medium and media with different deuterium content for 24h (left panel) and 72h (right panel). Deuterium concentration: control: D/H 150 ppm; No. 1: D/H 500.000 ppm; No. 2: D/H 100.000 ppm; No. 3: D/H 10.000 ppm; No. 4: D/H 75 ppm; No. 5: D/H 15 ppm. Phase-contrast microscopy. *↙*: stress fibers, *➝*: cell granularity, and **∗**: mitotic cells.

The morphology of ADSCs cultured in the control medium for 24 hours was characteristic for mesenchymal stromal cells: spindle-shaped fibroblast-like cells with a distinct nucleus, nucleoli, and perinuclear granularity [19]. Along with spindle-shaped fibroblast-like cells, the rounded dividing cells were also present (Figure 2, left panel). ADSCs in experimental media with different deuterium content were characterized by prominent morphological heterogeneity. Thus, in ADSCs cultured in media No. 1-5, a number of large cells with decreased nuclear-cytoplasmic ratio, granularity, and the presence of stress fibers were observed (Figure 2, left panel). The most pronounced ADSCs morphology changes were found after cultivation in a deuterium-rich growth medium (No. 1) (Figure 2, left panel). About 90% of the cell population was characterized by a decreased nuclear-cytoplasmic ratio, increased perinuclear granularity and the presence of stress fibers. Moreover, the cell density was significantly lesser compared with the control group and other experimental groups, and dividing cells were practically absent (Figure 2, left panel). Importantly, after 24 h of ADSCs cultivation in growth medium No. 4, where the deuterium content was closest to the natural one, the cells did not differ morphologically from the control culture (Figure 2, left panel).

After 72 h, the ADSCs in control medium formed a homogeneous fibroblast-like cell culture with 80-90% confluence (Figure 2, right panel). ADSCs cultured for 72h in experimental media (except No. 1) also reached confluence but preserved morphological heterogeneity (Figure 2, right panel). After 72h in culture ADSCs in deuterium-rich medium No. 1 (500.000 ppm D/H) did not reach confluence, they had polygonal morphology with even more pronounced stress fibers (Figure 2, right panel). Thus, the detected ADSCs morphological changes in growth media with different deuterium content indirectly indicate the deuterium influence on the ADSCs viability and proliferation rate.

### 3.3. ADSCs Cell Population Doubling Time (PDT) in Media with Different Deuterium Content

The ADSCs PDT had a tendency to increase in all experimental media compared to the control (Table 2). However, the statistically significant difference was only revealed for the ADSCs cultivated in media No. 1 and 2 with the highest deuterium content D/H 500.000 ppm and D/H 100.000 ppm. Since the PDT serves as one of the main cell proliferation activity indicators [20], its increase in the ADSCs population indicates cell cycle slowdown and, as a consequence, a decrease in the cell proliferation rate at high deuterium levels in the growth medium.

### 3.4. Deuterium Cytotoxicity in ADSCs Cell Cultures

**Figure 3:**
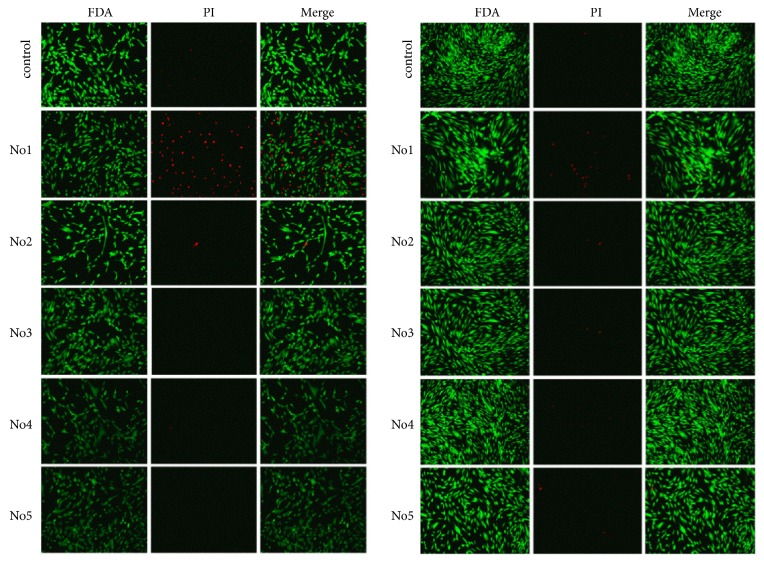
ADSCs in control and experimental media in 24h (left panel) and 72h (right panel) in culture. Deuterium concentration: control: D/H 150 ppm; No. 1: D/H 500.000 ppm; No. 2: D/H 100.000 ppm; No. 3: D/H 10.000 ppm; No. 4: D/H 75 ppm; No. 5: D/H 15 ppm. Fluorescent microscopy. FDA/PI staining.

The cytotoxicity was assessed with FDA and PI staining of ADSCs cultured in media with different deuterium content for 24h and 72h (Figure 3; Table 3).

The highest cell viability was observed in control medium with natural D/H ratio which was comparable with the deuterium-depleted medium (group No. 4). Reliably high deuterium toxicity was detected in the most deuterated growth medium (D/H 500.000 ppm). In the medium No. 2, the deuterium toxicity was delayed in time and manifested only after 72 hours of cultivation, which may indicate chronic toxicity. At the same time, the ADSC viability after 72 hours of cultivation in medium No. 1 was higher than in 24 hours, which indicates acute deuterium toxicity.

### 3.5. ADSCs Migration Ability in Growth Media with Different Deuterium Content (*In Vitro* Scratch Assay)

**Figure 4:**
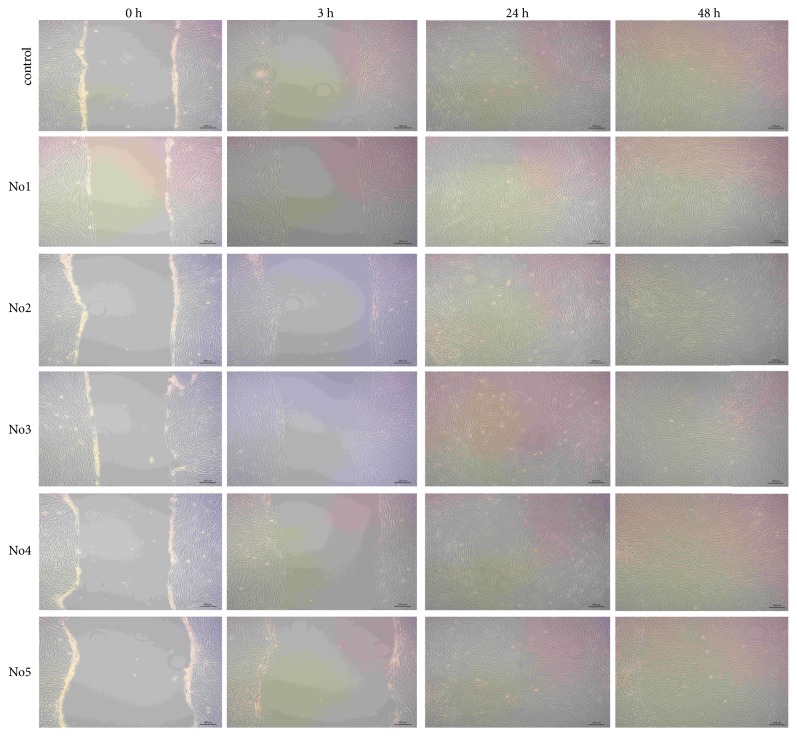
*In vitro* scratch assay with use of ADSCs in growth media with different deuterium content. Phase-contrast microscopy. Deuterium concentration: control: D/H 150 ppm; No. 1: D/H 500.000 ppm; No. 2: D/H 100.000 ppm; No. 3: D/H 10.000 ppm; No. 4: D/H 75 ppm; No. 5: D/H 15 ppm.

**Figure 5:**
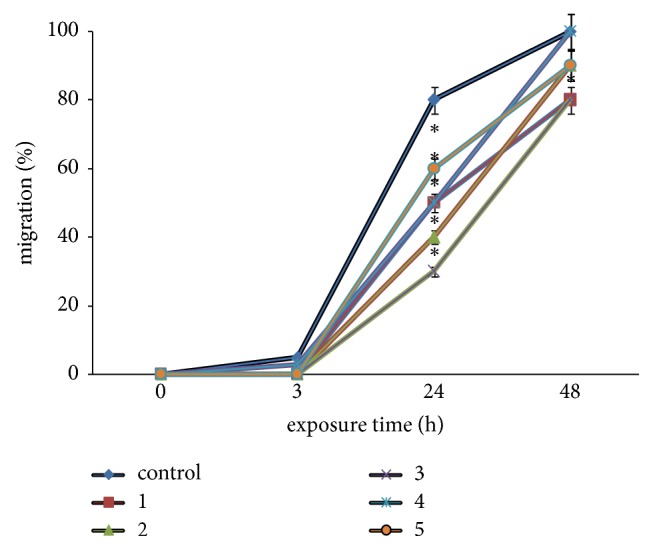
*In vitro* scratch assay with use of ADSCs for migration rate dynamics study in growth media with different deuterium content. Deuterium concentration: control: D/H 150 ppm; No. 1: D/H 500.000 ppm; No. 2: D/H 100.000 ppm; No. 3: D/H 10.000 ppm; No. 4: D/H 75 ppm; No. 5: D/H 15 ppm. The results are expressed as mean ± SD (n=6); *∗*: significant differences between the experimental group and the control, p<0.05.

Systemic administrated cultured mesenchymal stromal cells (MSC), including ADSCs, have the ability to migrate towards the damaged organs and tissues, contributing to regeneration [21]. The MSC migration ability depends on the expression profile of the chemokine and cytokine membrane receptors and adhesion molecules, as well as the cytokine status of the organism. It was shown that the MSC migration and homing activities can be artificially induced by the administration of high doses of granulocyte-macrophage colony stimulating factor and stromal cell-derived factor-1 [22]. The scratch assay is a classic method to assess cell migratory ability* in vitro*. We have used it to study the deuterium influence on the ADSCs migration. In vitro scratch assay was measured at 3, 24, and 48 hrs after scratching (Figures 4-5).

*In vitro* scratch assay in ADSCs culture in growth medium No. 4 (D/H 75 ppm) has shown the beginning of cell migration 3h after scratch. The most prominent difference in ADSCs migration ability was revealed 24h after scratch. Thus in growth media with high deuterium content (No. 1-3 - D/H 500.000–10.000 ppm) there was the slowest cell migration rate compared to control medium. Significant ADSCs migration delay was also observed in deuterium-depleted growth medium No. 5.

### 3.6. ADSCs Metabolic Activity in Growth Media with Different Deuterium Content

The Alamar Blue test system is based on the resazurin reduction to resorufin by metabolically active living cells. This is an intracellular conversion performed by mitochondrial, microsomal, and cytosolic oxidoreductases. Resazurin is nontoxic to cells and stable in growth media which allows investigating the cell proliferation rate and performing* in vitro *cytotoxicity tests with different compounds. Thus Alamar Blue is an integrative index of cellular metabolic activity which is determined by plasma membrane integrity, glycolysis intensity, mitochondria respiratory chain and synthesis processes activity [23].

Alamar Blue assay was performed after 24h and 72h of ADSCs cultivation in control or experimental growth media with different deuterium content.

**Figure 6:**
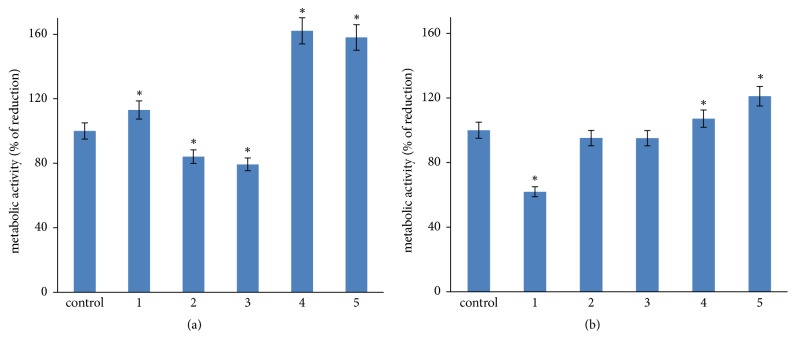
ADSCs metabolic activity in control and experimental groups in 24h (a) and 72h (b) in culture. Deuterium concentration: control: D/H 150 ppm; No. 1: D/H 500.000 ppm; No. 2: D/H 100.000 ppm; No. 3: D/H 10.000 ppm; No. 4: D/H 75 ppm; No. 5: D/H 15 ppm. The results are expressed as mean ± SD (n=6); *∗*: significant differences between the experimental group and the control, p<0.05.

There was a metabolic activity decrease at 24 h in ADSCs cultures in deuterated growth media (No. 2-3). Interestingly, there was an increase in ADSCs metabolic activity in 24 h in culture in deuterium-depleted growth media (Figure 6(a)). However, in 72 h in culture their metabolic activity was comparable to the control (Figure 6(b)). A reliable decrease in ADSCs metabolic activity was observed in 72 h in culture in growth medium with maximum deuterium content D/H 500.000 ppm which could evidence the deuterium cumulative effect and chronic toxicity.

## 4. Discussion

In living systems, hydrogen plays a direct role in oxidation-reduction processes within the cell, participating as a donor and acceptor in various biochemical reactions. Therefore, a change in the deuterium/protium isotope ratio can lead to both acceleration and retardation of such reactions regarding specific features of the protium-deuterium bonds. It was shown that deuterium bonds are more durable and their rupture is more energy-consuming compared to protium [24, 25]. In organic compounds that are part of living tissues, deuterium water is more stable and inactive, so it is not included in the metabolic processes and inhibits them [4, 13]. Our data confirm isotope effects, because in all concentrations of deuterium above natural values, there was a decrease in the time of population doubling, viability, cell migration rate, and metabolic activity in cultures, as well as a change in morphological features indicative of the stress effects of the deuterated medium. At the same time, at lower deuterium concentrations in the growth medium, there was an increase in the migration rate and metabolic activity compared to control.

In the deuterated growth medium, we observed stress fibers and increase in cell size compared to control (Figure 2), accompanied by decrease of proliferation and metabolic activity and senescence of a cell culture with conversion ADSCs into myofibroblast-like phenotype [25]. The above-mentioned effect was also detected in the deuterium-depleted growth medium. The ADSCs population doubling time increased in deuterated water which indicated a decrease of cell proliferation rate. This was also previously shown on tumor cell lines [25–27]. Hence, it can be concluded that a nutrient medium with deuterium content close to natural values is the most optimal for systems* in vitro*, which should be taken into account when investigating the deuterium action for medical and therapeutic purposes in systems* in vivo*.

The data on the ADSCs viability at 24 h and 72 h indicate that cell cultures are able to adapt to the replacement of protium by deuterium (at 24 h the acute toxicity in group No.1 is observed (Table 3), although the level of chronic toxicity remains high at 72 h). Interestingly, in deuterium-depleted medium (No. 5) chronic toxicity appeared at 72 h (Table 3). These results can be explained by the suggestion that certain amounts of deuterium in growth medium are necessary to inhibit the processes associated with cell metabolic activity [5, 10]. Thereby, both deficiency and excess of deuterium could be negative for cell activity in vitro (also described by other authors [28, 29]).

It should be noted that the study of the deuterium properties as a component of living systems is not limited by only its biological role determination [30]. There is a theory of density inhomogeneities in water with different deuterium content, which suggests that deuterium, depending on its concentration, acts as a control element of various physicochemical properties of water [31]. According to this theory, exchange processes, diffusion, etc., have higher reaction kinetics in deuterium-depleted water [32]. Our data are supported by this theory in a way that in the absence of density inhomogeneities in deuterium-depleted water, metabolic activity as a whole is intensified. And, conversely, in deuterated water, all processes are slowed down not only from the biological point of view within the cell, but also from the point of view of increasing the nonuniformity of water density, which slows down metabolic processes in the environment outside the cell [33]. Our data correlate with the data of other authors, where similar effects of water of different D/H isotope composition are observed in different systems* in vitro* and* in vivo* [34–36].

The data from Alamar Blue assay are discussable as ADSCs metabolic activity after 24 h in culture in the medium with highest deuterium content (No. 1, Figure 6(a)) is comparable with control medium. This effect can be explained by the fact that deuterium is slowly incorporated into biomolecular structures inside the cell [4, 37], so at the 24 h stage there is no substitution of protium for deuterium in those complexes that are responsible for the transformation of resazurin. The main centers that transform resazurin into resorufin are located in mitochondria. Perhaps the first replacement of protium by deuterium was realized in the cytoplasmic metabolic signaling pathways, not affecting the mitochondria, which led to a visible change in the ADSCs morphology. Restoration of resazurin continued with the same intensity in the mitochondria for 24 hours of the experiment, although the remaining cycles, where deuterium was already present, were inhibited. At 72 h of the experiment, deuterium was apparently integrated into all complexes and basic biomolecules, including mitochondria, which was reflected in a sharp metabolic activity decrease in the most deuterated media.

We should also consider the adaptation of cell cultures to the changed deuterium isotope composition in the growth medium. As can be seen from the cytotoxicity and metabolic activity assessment, in many comparison groups, except control, an increase in the indicator values are observed at 24 h of the experiment and a decrease in 72 h. This effect could also be considered as the so-called shock (functional) effect [28, 38–41] when the culture is transferred from one growth medium to another. However, redistribution of deuterium and protium in intracellular structures probably leads to a gradual adaptation of the culture to the new D/H isotope ratio. Over time, this is reflected in a decrease in cytotoxicity and increase in metabolic activity which enables the proliferation of the culture, which is confirmed by the migration ability in extreme comparison groups.

The obtained data give evidence of the direct role of deuterium in cell culture* in vitro*. These results can be used in biomedical and therapeutic studies, where deuterium can be considered as a regulator of the biological properties of normal and/or cancer cells. However, the deuterium role in complex biological systems has not been fully established, which requires further studies in the IVIVC system. The effect of deuterium in water on biological objects of different hierarchic levels has not only theoretical, but also important practical applications. First of all, that concerns the medicine. It is well known that one of the pharmaceutical trends is the development of deuterium-containing drugs, which are deuterated analogues of already known proton-containing biologically active compounds [10, 11, 29, 34, 42]. The second direction refers to the role of deuterium-depleted water as an adjuvant in the treatment of cancer [12, 13]. The result of the change in D/H ratio is manifested in the form of kinetic isotopic effect, which is characterized by a change in the rate of absorption, distribution, biotransformation and excretion of the medicines. Moreover, the development of methodological approaches to drugs quality control based on water isotopologues could improve pharmaceutical analysis and optimize their dosages, reducing the toxic load on the body.

## 5. Conclusion

Our data indicate that deuterium plays an important role in intracellular processes that are responsible for the metabolism and viability of ADSCs cultures* in vitro*. We have observed a change in practically all indexes in the increased deuterium content conditions in the growth medium. Under deuterium content below natural there was a stimulating effect on proliferation activity, but deuterium depletion caused cytotoxicity.

## Figures and Tables

**Table 1:** Immunophenotype of human ADSCs.

Cell culture no. / CD marker	СD90	CD105	CD73	CD34	CD45	HLA-DR
No1	95,7%	98,6%	99,6%	1,2%	0,1%	0,9%
No2	90%	92,6%	94,6%	1,4%	0,9%	0,9%
No3	97,8%	94,3%	97,9%	0,6%	0,2%	0,9%
No4	98,2%	98,4%	98,4%	0,8%	0,2%	0,6%
No5	95,5%	97,65	97,5%	0,6%	0,4%	0,5%
Mean±SD	95,44±3,3%	96,3±2,7%	97,6±1,9%	0,92±0,4%	0,36±0,3%	0,76±0,2%

**Table 2:** ADSCs PDT in growth media with different deuterium content. The results are expressed as mean ± SD (n=6), significant differences between the experimental group *∗*p<0.05 compared to control group; #p<0.05 compared to groups No. 2-5.).

	Control D/H 150 ppm	No. 1 D/H 500.000 ppm	No. 2 D/H 100.000 ppm	No. 3 D/H 10.000 ppm	No. 4 D/H 75 ppm	No. 5 D/H 15 ppm
PDT, hours	31.91±1.67	56.24±1.92^*∗*,#^	41.34±1.58^*∗*^	36.34±1.30	32.42±1.92	33.43±1.58

**Table 3:** ADSCs viability in growth media with different deuterium content. The results are expressed as mean ± SD (n=6), significant differences between the experimental group *∗*p<0.05 compared to control, #p<0.05 compared to group No. 1, and ##p<0.05 compared to groups No. 2-5.).

Viability, %	Control D/H 150 ppm	No. 1 D/H 500.000 ppm	No. 2 D/H 100.000 ppm	No. 3 D/H 10.000 ppm	No. 4 D/H 75 ppm	No. 5 D/H 15 ppm
24h	97.82±1.30	50.80±5.11^*∗*,##^	91.61±3.78	91.21±2.48	97.03±1.58	92.24±1.92
72h	97.62±1.14	75.41±6.10^*∗*,##^	85.03±3.16^*∗*,#^	91.25±3.19	96.27±1.30	90.81±1.30*∗*

## Data Availability

The data used to support the findings of this study are available from the corresponding author upon request.
